# In Response to the Letter by Nevin

**DOI:** 10.4269/ajtmh.18-0771b

**Published:** 2019-02

**Authors:** Aaron I. Schneiderman, Yasmin S. Cypel, Erin K. Dursa, Robert M. Bossarte

**Affiliations:** Department of Veterans Affairs, Epidemiology Program; Post Deployment Health Services (10P4Q), Office of Patient Care Services, Veterans Health Administration; Washington, District of Columbia; E-mail: aaron.schneiderman@va.gov; Department of Behavioral Medicine and Psychiatry; West Virginia University Injury Control Research Center, West Virginia University School of Medicine; Morgantown, West Virginia

Dear Sir,

In a letter to the editor^1^ commenting on our article^2^ on the association between antimalarial medications (including mefloquine) and health outcomes in veterans, three concerns with our analyses were presented. We disagree with the premise that the stated concerns diminish our findings as they are largely theoretical and lack empirical support.

The first concern addressed the magnitude of the observed (unadjusted) association between self-reported mefloquine exposure and mental health outcomes. The author asserts that the reported relationship reflects the influence of information bias, specifically differential misclassification of a more appropriate exposure of interest, in this case the presence of prodromal symptoms after taking mefloquine. The author contends that combining mefloquine users with and without acute side effects “results in a biased, unadjusted, effect toward the null.” We disagree with this contention that the acute presentation of side effects, or prodromal symptoms, is a more accurate measure of exposure when considering the association between mefloquine use and chronic mental health outcomes. A 2017 Cochrane systematic review of the safety and efficacy of mefloquine for malaria prophylaxis cites drug manufacturer labels that suggest side effects may be prodromal and dose related.^3^ Although they cite evidence for acute side effects in cohort studies, randomized controlled trials, and retrospective analyses, the review states that evidence of long-term statistically significant effects of mefloquine is limited.^3^ Although worthy of consideration and further study, we do not find sufficient evidence to support the possibility that misclassification bias has significantly influenced findings reported in our article.

The second concern stated that the “multivariate regression on covariates correlated with this exposure (unmeasured presentation of acute side effects) has resulted in unrecognized confounding.” The author suggests that combat and deployment variables are correlated with the unmeasured presentation of acute side effects of mefloquine that are postulated to be causally related to chronic neuropsychiatric effects. To our knowledge, this hypothetical assertion is not supported by existing studies. This assertion supposes that a subgroup of persons experiencing prodromal symptoms have increased vulnerability to symptoms consistent with mental health disorders independent of the effects of other traumatic events associated with those same outcomes. The presence of underlying vulnerability to long-lasting symptomology does not exclude the possibility that expression of that vulnerability is a function, at least in part, of exposure to additional stressors (i.e., combat). The inclusion of both mefloquine administration and combat exposure in fully adjusted models allows for consideration of the effects associated with each exposure.

It is further argued by Dr. Nevin that military command–directed administration of mefloquine is associated with both combat exposure and continued drug administration in the presence of potentially harmful side effects and may be an intermediate variable in a causal pathway between drug administration and chronic illness. This argument is purely conjecture and not supported by scientific evidence. The determination of causality is multifaceted and dependent on factors including experimental evidence (typically defined through the conduct of randomized clinical trials to guard against confounding and bias) and temporality (in this case whether mefloquine exposure preceded outcomes of interest). These factors have not been appropriately considered by Dr. Nevin.

Third, we disagree with Dr. Nevin’s interpretation of our conclusions and the statement that the effects of combat exposure on mental health burden are not supported by our data. We concluded, “While there appeared to be significant elevated odds of poor mental and physical health outcomes among those who reported antimalarial use relative to nonusers, once the effect of combat exposure was adjusted for, significant relationships generally diminished, implying that in this large population-based sample, it is the effect of combat exposure that is driving the mental health burden, not the exposure to antimalarial medication.”^2^ The factors we adjusted for in these analyses are those that are well recognized (i.e., deployment and combat exposure) as potent predictors of mental health outcomes in veterans.^4,5^

The author’s contention regarding our conclusion is that the unadjusted results show an association between mefloquine use and mental health outcomes and that this is an indication that a relationship exists, because we failed to address certain “unrecognized” confounders. Unadjusted findings typically introduce potential relationships that need to be tested by statistically controlling for pertinent covariates. In this case, after controlling for combat exposure and deployment, we found that the associations between mefloquine and mental health outcomes were not supported by multivariable regression analyses. Our findings on the association between combat exposure and mental health are supported by other research on military and veteran populations^4,5^; additional evidence for this association is presented in Table 5 of our article, where a monotonic relationship is observed between the level of self-reported combat intensity and the prevalence of screening positive for mental health symptoms.^2^ Although some may suggest that regression analysis of cross-sectional data can help to infer causal associations between a dependent variable and selected independent variables, it is a widely acknowledged tenet in epidemiologic research that any reference to causal associations be viewed cautiously to minimize claims of direct associations that may be spurious in nature.

In summary, we believe Dr. Nevin’s comments^1^ on our article^2^ are unfounded. The strengths and limitations of our study are noted in the Discussion section. This was a cross-sectional survey that can only assess associations and does not establish causality. Our analyses controlled for recognized confounders, and although there is always the possibility of unmeasured confounding, it is yet to be determined whether further adjustments would reverse or negate the observed associations. In the absence of a randomized controlled trial of antimalarial medication use to assess the development of chronic health outcomes, causal statements regarding the effect(s) of mefloquine use on long-term neuropsychiatric symptoms should be viewed with caution.
